# A feasibility randomised controlled trial of water-only fasting prior to CAPOX chemotherapy for stage 2/3 colorectal cancer

**DOI:** 10.1186/s40814-026-01848-0

**Published:** 2026-06-03

**Authors:** Georgia Herbert, Clare England, Ellie Shingler, Rachel M. Barker, Claire M. Perks, Aidan Searle, Alex Whitmarsh, Andy Ness, Mark Saunders, Charlotte Atkinson

**Affiliations:** 1 NIHR Bristol Biomedical Research Centre (Nutrition Theme), University Hospitals Bristol and Weston NHS Foundation Trust and University of Bristol, Bristol, UK; 2 Cancer Endocrinology Group, Learning & Research Building, Bristol Medical School, Southmead Hospital, Translational Health Sciences, Bristol, BS10 5NB UK; 3 The Christie NHS Foundation Trust, Wilmslow Road, Manchester, M204BX UK

**Keywords:** Fasting, Chemotherapy, Colorectal cancer, CAPOX, Feasibility trial

## Abstract

**Background:**

Capecitabine and Oxaliplatin (CAPOX) chemotherapy is a standard treatment for stage 2/3 colorectal cancer but is associated with dose-limiting toxicities. Short-term fasting may protect healthy cells from chemotherapy-related side effects via differential stress resistance. However, it is not known whether it is possible to recruit to a trial of short-term fasting in this population, or whether participants would adhere to the intervention. The aim was to test the feasibility and acceptability of a pre-chemotherapy, 36-h, water-only fast prior to the first three cycles of chemotherapy in people receiving CAPOX chemotherapy for stage 2/3 colorectal cancer.

**Methods:**

A two-armed randomised controlled feasibility trial with an embedded qualitative interview study conducted in two NHS foundation trust hospital sites in England. Participants were randomly allocated, in a 1:1 ratio using random permuted blocks, by a secure online randomisation system, to either a 36-h fast immediately prior to chemotherapy administration or standard dietary advice. The intervention was delivered during chemotherapy cycles 1–3. Primary outcomes were adherence, recruitment, retention, data completion, and acceptability. Secondary outcomes included chemotherapy side effects, quality of life, metabolic and inflammatory markers, appetite, and sarcopenia.

**Results:**

Seventy-nine patients were screened, of whom44 were eligible. Twelve consented and were randomised (intervention: *n* = 6; control: *n* = 6). The retention rates for chemotherapy cycles 1, 2, and 3 were 100%, 83%, and 83% for the intervention group and 100%, 100%, and 100% for the control. Five out of six participants adhered to the fast. The trends observed in levels of insulin-like growth factor I (IGF-I) and insulin-like growth factor binding proteins 2 and 3 (IGFBP-2 and IGFBP-3) were consistent with effective fasting. Qualitative findings indicated that fasting was achievable but sometimes perceived as an additional burden alongside chemotherapy. Recruitment challenges were influenced by site-level factors, including competing priorities, COVID-19-related disruptions, and perceptions of the intervention.

**Conclusion:**

The SWiFT study was feasible to deliver and adherence to the fast was high. In a future definitive trial, success in recruitment appears to depend more on specific local factors rather than the intervention being unattractive to most potential participants.

**Trial registration:**

ISRCTN, ISRCTN17994717, registered 23 October 2018, https://www.isrctn.com/ISRCTN17994717

**Supplementary Information:**

The online version contains supplementary material available at 10.1186/s40814-026-01848-0.

## Key messages regarding feasibility

- What uncertainties existed regarding the feasibility?
  The aim of the Short-term, Water-only Fasting Prior to Chemotherapy Trial (SWiFT) feasibility study was to examine the feasibility and acceptability of a water-only 36-h fast prior to CAPOX chemotherapy for stage 2/3 colorectal cancer.
- What are the key feasibility findings?
  Site recruitment was challenging and a greater emphasis on engagement is required at the set-up phase. SWiFT confirmed that a 36-h fast prior to CAPOX chemotherapy was possible. The SWiFT study was acceptable to participants and feasible to implement in practice and adherence to the 36-h fast was high.
- What are the implications of the feasibility findings for the design of the future trial?
  The recruitment strategy was feasible without disrupting routine clinical care and was considered a straightforward design with low impact data collection. However engaging sites for recruitment may be a challenge due to competing trials and research priorities, concerns about fasting as an additional burden for patients, and wider disruptions during the study period. These findings highlight the importance of early site engagement, clear communication of the intervention rationale, and support for recruitment processes. Future studies can use the findings from this study to refine the features and delivery of a fasting intervention.

## Background

Colorectal cancer is the fourth most common cancer in the UK, with 41,300 new cases diagnosed each year [1]. A standard treatment in the UK for stage 3 colorectal cancer (or stage 2 colorectal cancer that is at high risk of local recurrence) is surgical resection, followed by Capecitabine and Oxaliplatin (CAPOX) chemotherapy [2]. As well as affecting patient experience and quality of life, dose-limiting toxicities (defined as severe or prolonged side effects which limit or delay subsequent doses of systemic therapies [3, 4]) may reduce treatment efficacy. It has been shown that around 57% of people receiving CAPOX required dose modifications due to toxicity [5].

Dietary restriction at the time of chemotherapy has been suggested as a way to potentially attenuate the cytotoxic effects of cancer treatments such as chemotherapy and radiotherapy [6], as some pre-clinical studies have shown differences between cancer cells and non-cancer cells in their response to dietary restriction that may, in turn, affect response to chemotherapy. Fasting causes non-tumour cells to alter their metabolic pathways through a complex cell signalling process to conserve energy for maintenance and repair, such as reduced cell division and increased autophagia. This allows organisms to maximise their chance of survival by diverting energy for repair during periods of nutrient deprivation [7] and is partially mediated by a reduction in glucose and insulin-like growth factors (IGFs) [7, 8]. However, two of the recognised hallmarks of cancer are the ability of tumour cells to continue to grow in the absence of growth factors and in the presence of growth inhibitory signals [9]. The genetic changes associated with tumour cell development, such as loss-of-function mutations in tumour suppressor genes (e.g. p53, PTEN) that leads to insensitivity to growth inhibitory signals, and gain of function mutations in oncogenes (e.g. Ras, Akt, and mTOR), culminates in proliferation pathways continually being active, even in the absence of growth signals [10]. Therefore, tumour cells do not respond to nutrient deprivation in the same way as non-tumour cells and continue to proliferate, even when nutrients are scarce. This could potentially reduce their ability to protect and repair themselves in the absence of nutrient supply.

These differences in reaction to nutrient scarcity between non-cancer and cancer cells collectively lead to a concept which has been referred to as differential stress resistance (DSR) whereby tumour cells may be more susceptible to stress induced by cytotoxic treatments, such as chemotherapy, than non-tumour cells. Although the pre-clinical studies have shown promising results [6–8], very few studies have been conducted in humans, and it is unclear how well the findings from pre-clinical studies translate to humans, and whether such interventions are even feasible in people undergoing cancer treatment.

Several different types of dietary restriction have been considered as possible therapeutic strategies including short-term fasting, fasting mimicking, ketogenic, protein restriction, and time-restricted diets [11]. In a recent scoping review, four studies of fasting at the time of chemotherapy in humans were identified including a pilot RCT in 13 people with breast cancer [12] and a dose escalating safety and feasibility trial in 20 people receiving platinum-based chemotherapy for any cancer type [13]. The studies showed that some people were willing to fast for up to 72 h, although adherence tended to be poorer than for shorter fasts (24 h or 48 h). There were some differences in haematologic toxicities between the fasting and control groups, but the studies were not powered to detect differences and must be interpreted with caution. Other published studies of fasting at the time of chemotherapy in humans included a qualitative study [14] and a case series report [15].

To our knowledge, no studies to date have looked at fasting at the time of chemotherapy as a potential way to reduce toxicities in colorectal cancer patients being treated with CAPOX chemotherapy. The aims of this study are to test the feasibility of a short-term fasting intervention in people undergoing chemotherapy for colorectal cancer and to explore participant acceptability of taking part in the trial through an embedded qualitative interview study.

## Methods

The protocol for the SWiFT has previously been published [16], but the methods are briefly described below, along with changes made since publication of the protocol and other pertinent information.

### Participant recruitment and randomisation

SWiFT opened for recruitment at two NHS hospitals in March 2020 [The Christie NHS Foundation Trust and University Hospitals Bristol and Weston NHS Foundation Trust (UHBW)] but closed shortly thereafter due to the COVID-19 pandemic. In July 2020 and April 2021, SWiFT re-opened for recruitment at The Christie and UHBW, respectively. Participant recruitment continued until April 2022, and all participants were recruited from the Christie NHS Foundation Trust, with none being recruited from UHBW.

#### Eligibility criteria

Participants were eligible for the study if they were aged ≥ 18 years; had histologically confirmed stage 2/3 colorectal cancer being treated with adjuvant CAPOX chemotherapy; had a performance status ≤ 2; and were able to provide written informed consent. Participants were ineligible if they had confirmed cachexia; were on medication for diabetes; had a body mass index (BMI) ≤ 18.5 kg/m^2^; had a history of an eating disorder; had a recent history of drug or alcohol abuse; were participating in another study that may affect the outcomes of this feasibility trial; or were unable to speak/understand English.

#### Recruitment

Potential participants were identified through multidisciplinary team (MDT) meetings and screening of clinic lists by the site trial team. Following the MDT, they were provided with verbal and written information about the trial by a member of their usual care team. Those who wished to enrol were formally assessed for eligibility at a screening visit.

#### Randomisation and blinding

Eligible and interested individuals were asked to provide written informed consent, after which they were randomised with full allocation concealment to either a 36-h fast or usual care as per local standard practice. Usual care included standard dietary advice provided as part of routine clinical care during chemotherapy. Due to the nature of the intervention, blinding of participants was not possible, but biological samples were analysed without knowledge of treatment allocation.

### Trial arms

Participants randomised to the intervention arm were asked to undertake a 36-h water-only fast, immediately prior to chemotherapy administration in cycles 1–3. If needed, they were allowed to consume small amounts of food to mitigate any side effects of fasting and were provided with a list of 50 kcal snacks. Participants were considered to have adhered to the fast if they consumed less than 14% of their basal metabolic rate (BMR) requirements (kcal/day) calculated using the Oxford equations for BMR, in the 36 h prior to chemotherapy administration. This equates to approximately 200 kcal per day, depending on age and gender.

Participants randomised to the control arm received usual care as per local standard practice.

### Sample size

As this is a feasibility trial, a formal power calculation was not performed and a sample size of 30 was chosen (15 participants at each site), based on the recommended sample size required to estimate key parameters for primary outcomes [17, 18]. These estimates will subsequently inform power calculations for future definitive trials.

### Data collection and outcome measures

#### Outcome measures

The eligibility rate was calculated by dividing the number of people screened for eligibility by the number who met inclusion criteria. The outcome measures have been described in detail previously (Shingler et al. NaN, NaN). Data were collected from participants (via questionnaires) and their medical records. Briefly, the primary outcomes were recruitment rate; retention rate; adherence to intervention; acceptability and tolerability of the intervention; and data completion rates. Formal progression criteria (e.g. stop/go thresholds) were not pre-specified, as the aim of this feasibility study was to estimate key feasibility parameters and explore acceptability to inform the design of a future definitive trial. Secondary outcomes were side effects of chemotherapy; quality of life; haematologic toxicities; markers of cellular metabolism; markers of inflammation; appetite; and hand grip strength.

In a deviation from the published protocol, sarcopenia was not assessed from computerised tomography (CT) scans because they were completed 6 months post-surgery, which resulted in a large time interval between the end of the intervention and the follow-up CT scan.

#### Qualitative interviews

After completing the trial, participants were invited to take part in a semi-structured interview conducted via telephone. Interview questions focused on experiences regarding participation in the intervention and data collection processes [19]. As an addition to the published protocol, we received ethics approval to interview the personnel involved in recruitment at the two study sites in order to understand factors affecting recruitment.

### Blood sample analysis

Routinely collected full blood counts and blood chemistry data were obtained from samples collected when the patient attended their screening visit and prior to the second and third chemotherapy cycles. Study specific blood samples obtained immediately prior to chemotherapy administration at cycles 1 and 3 were analysed for glucose, insulin, C-reactive protein (CRP), IGF-I, IGF-II, IGFBP-2, and IGFBP-3. For assessment of IGFs, serum samples were transferred to the University of Bristol on dry ice and stored at − 80 °C. IGF-I, IGF-II, and IGFBP-3 levels were assessed using in-house radio-immunoassays as described previously [20]. Peptides labelled with iodine-125 were purchased from Generon, Slough, UK. Samples were assayed in triplicate and the average concentration used for analysis. IGFBP-2 levels were assessed using an ELISA (DY674, Bio-Techne, Minneapolis, USA) according to the manufacturer’s instruction, with the exception that the standard curve was prepared by diluting recombinant IGFBP-2 peptide (Gropep Bioreagents, Thebarton, Australia) in reagent diluent to give a concentration range of 15–0.3 ng/ml. Samples were assayed in duplicate, and the average concentration used for analysis.

### Dietary data

Participants in both arms were asked to complete food diaries for 36 h before each chemotherapy cycle. Food diaries were coded and analysed using Dietplan7 Pro dietary analysis software (Forestfield Software Limited) using standardised methods [21]. Reported energy intake for each participant was estimated.

### Data analysis

#### Quantitative data

Primary feasibility outcomes were recruitment, retention, and adherence rates, reported as percentages. Secondary outcome data were summarised using means (standard deviations) or medians (inter-quartiles ranges) as appropriate for continuous variables and frequencies with percentages (*n*, %) for categorical variables. All quantitative data were analysed in Stata 16 [22].

#### Qualitative data

Audio recordings of interviews with participants and research staff were transcribed verbatim and anonymised. The participant interviews were analysed thematically using the Framework method [23]. A deductive approach was taken to the analysis of the healthcare professional interviews where a checklist was used to organise the interview data to explore factors affecting recruitment into SWiFT. This approach was informed and guided by a checklist of reasons for recruitment failure developed from a systematic review of 172 RCTs discontinued due to poor recruitment and 49 interviews with clinical trial stakeholders [24]. Analysis was undertaken by GH and AS who double-coded 50% of transcripts to enhance reliability. The analysis was assisted by Nvivo 10 software.

## Results

**Fig. 1:**
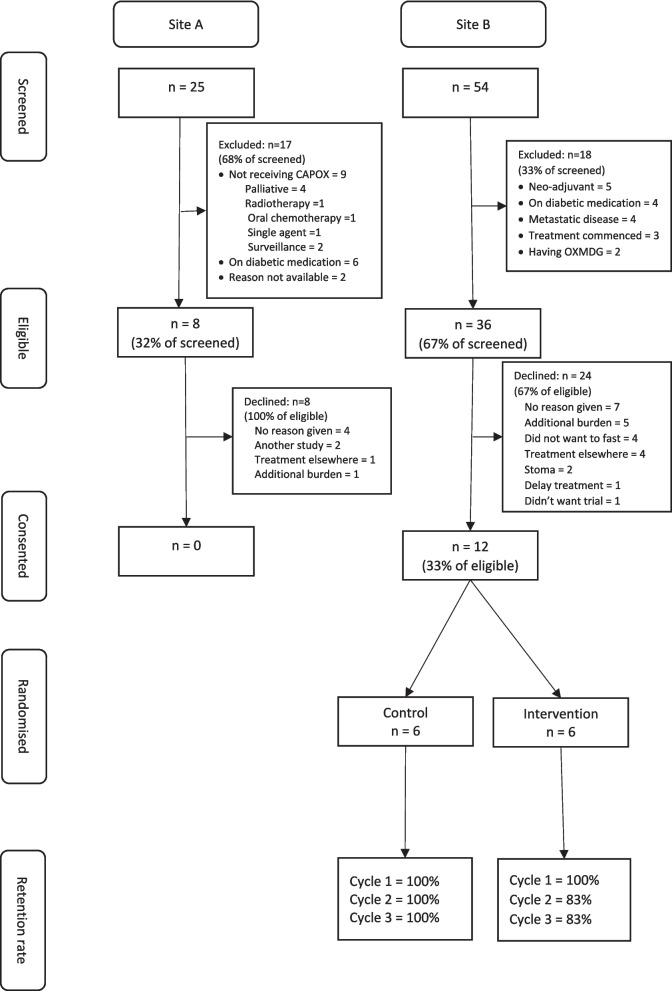
Flowchart of participants

Figure 1 shows the flow of participants throughout the trial and Table 1 shows the baseline demographic characteristics of all study participants, by treatment arm.

**Table 1:** Baseline demographic characteristics of all study participants, by treatment arm

Variable	Intervention (*n* = 6)	Control (*n* = 6)
Age (y), mean (SD)	63.2 (8.7)	58.2 (12.4)
Gender, *n* (%)		
Male	4 (66.7%)	2 (33.3%)
Female	2 (33.3%)	4 (66.7%)
TNM stage, *n* (%)		
I/II	0 (0%)	0 (0%)
III/IV	6 (100%)	5 (100%)
Weight (kg), mean (SD)	92.1 (14.5)	79.7 (18.1)
Height (cm), mean (SD)	172.3 (13.1)	170.0 (10.6)
Hand grip strength (kg), mean (SD)	32.6 (11.1)	23.3 (5.3)
Body mass index (kg/m^2^), mean (SD)	31.8 (2.9)	27.3 (4.4)
Basal metabolic rate (kcal/day), mean (SD)	1698.3 (254.5)	1537.3 (307.8)
ECOG performance status, *n* (%)		
Grade 0	5 (83.3%)	5 (83.3%)
Grade 1	1 (16.7%)	1 (16.7%)
Scheduled capecitabine dose (g/m^2^), *n* (%)		
1	1 (16.7%)	1 (16.7%)
50		1 (16.7%)
100	1 (16.7%)	3 (50%)
500	3 (50%)	
1000	1 (16.7%)	1 (16.7%)
Scheduled oxaliplatin dose (mg/m^2^), *n* (%)		
104	2 (33.3%)	0 (0%)
130	4 (66.7%)	6 (100%)

### Primary outcome measures

#### Eligibility rate and reasons for exclusion

The eligibility rate was 32% in site A and 67% in site B. Main reasons for exclusion were patients not receiving CAPOX chemotherapy (palliative, radiotherapy, oral chemotherapy, single agent) or on diabetic medication (Fig. 1).

#### Recruitment and retention rates

At site A, eight out of 25 people screened were deemed to be eligible and zero were recruited. At site B, 36 out of 54 people screened were deemed to be eligible and 12 people were recruited (33.3%). The retention rates for cycles 1, 2, and 3 were 100%, 83%, and 83% for the intervention group and 100%, 100%, and 100% for the control group (see Fig. 1).

#### Data completion rates

Twelve participants returned food diaries at cycle 1 and 11 participants for cycles 2 and 3. Completion rates for other measures are reported in Tables 2 and 3.

**Table 2:** Patient-reported outcome measures (PROMs)

	Intervention									Control								
	Cycle 1			Cycle 2			Cycle 3			Cycle 1			Cycle 2			Cycle 3		
	Day1	Day3	Day7	Day1	Day3	Day7	Day1	Day3	Day7	Day1	Day3	Day7	Day1	Day3	Day7	Day1	Day3	Day7
EQ-5D-5L score																		
Mean (SD)	0.93 (0.07) *N* = 6	0.88 (0.08) *N* = 6	0.87 (0.12) *N* = 6	0.93 (0.05) *N* = 6	0.90 (0.07) *N* = 5	0.90 (0.07) *N* = 6	0.95 (0.06) *N* = 5	0.89 (0.05) *N* = 6	0.86 (0.08) *N* = 6	0.96 (0.06) *N* = 6	0.83 (0.08) *N* = 5	0.79 (0.19) *N* = 6	0.89 (0.07) *N* = 4	0.62 (0.23) *N* = 4	0.82 (0.10) *N* = 5	0.94 (0.05) *N* = 4	0.78 (0.12) *N* = 5	0.83 (0.09) *N* = 5
Hunger VAS																		
Mean (SD)	64.2 (42.2) *N* = 6	41.5 (37.4) *N* = 4	55.8 (39.0) *N* = 6	60.0 (46.9) *N* = 4	51.3 (46.6) *N* = 4	41.0 (42.5) *N* = 5	71.3 (42.5) *N* = 4	53.6 (49.4) *N* = 5	53.8 (49.5) *N* = 5	35.5 (20.6) *N* = 6	52.5 (20.4) *N* = 6	51.2 (25.9) *N* = 6	48.3 (22.3) *N* = 6	45.0 (22.6) *N* = 5	55.5 (21.6) *N* = 6	55.7 (18.3) *N* = 6	52.5 (17.8) *N* = 6	54.2 (18.0) *N* = 6
Pro-CTCAE—*n* (%) experiencing grade 3 or 4																		
Swallowing (severity)	0 (0%)	–	0 (0%)	0 (0%)	–	0 (0%)	0 (0%)	–	0 (0%)	0 (0%)	–	1 (17%)	0 (0%)	–	1 (17%)	0 (0%)	–	1 (17%)
Mouth/throat sores (severity)	0 (0%)	–	0 (0%)	0 (0%)	–	0 (0%)	0 (0%)	–	0 (0%)	0 (0%)	–	0 (0%)	0 (0%)	–	0 (0%)	0 (0%)	–	0 (0%)
Mouth/throat sores (interfere)	0 (0%)	–	0 (0%)	0 (0%)	–	0 (0%)	0 (0%)	–	0 (0%)	0 (0%)	–	0 (0%)	0 (0%)	–	0 (0%)	0 (0%)	–	0 (0%)
Tasting (severity)	0 (0%)	–	0 (0%)	0 (0%)	–	0 (0%)	0 (0%)	–	0 (0%)	0 (0%)	–	0 (0%)	0 (0%)	–	0 (0%)	0 (0%)	–	0 (0%)
Appetite (severity)	0 (0%)	–	1 (17%)	1 (25%)	–	1 (20%)	1 (25%)	–	1 (20%)	0 (0%)	–	0 (0%)	0 (0%)	–	0 (0%)	0 (0%)	–	0 (0%)
Appetite (interfere)	0 (0%)	–	1 (17%)	0 (0%)	–	0 (0%)	1 (25%)	–	0 (0%)	0 (0%)	–	0 (0%)	0 (0%)	–	0 (0%)	0 (0%)	–	0 (0%)
Nausea (frequency)	0 (0%)	–	1 (17%)	0 (0%)	–	1 (20%)	0 (0%)	–	1 (20%)	0 (0%)	–	1 (17%)	0 (0%)	–	1 (17%)	0 (0%)	–	1 (17%)
Nausea (severity)	0 (0%)	–	1 (17%)	0 (0%)	–	0 (0%)	0 (0%)	–	0 (0%)	0 (0%)	–	0 (0%)	0 (0%)	–	0 (0%)	0 (0%)	–	0 (0%)
Vomiting (frequency)	0 (0%)	–	0 (0%)	0 (0%)	–	0 (0%)	0 (0%)	–	0 (0%)	0 (0%)	–	0 (0%)	0 (0%)	–	0 (0%)	0 (0%)	–	0 (0%)
Vomiting (severity)	0 (0%)	–	0 (0%)	0 (0%)	–	0 (0%)	0 (0%)	–	0 (0%)	0 (0%)	–	0 (0%)	0 (0%)	–	0 (0%)	0 (0%)	–	0 (0%)
Constipation (severity)	0 (0%)	–	0 (0%)	0 (0%)	–	0 (0%)	0 (0%)	–	1 (20%)	0 (0%)	–	0 (0%)	0 (0%)	–	1 (17%)	0 (0%)	–	1 (17%)
Diarrhoea (frequency)	0 (0%)	–	0 (0%)	1 (25%)	–	0 (0%)	0 (0%)	–	0 (0%)	0 (0%)	–	2 (33%)	1 (17%)	–	1 (17%)	0 (0%)	–	1 (17%)
Abdomen pain (frequency)	0 (0%)	–	1 (17%)	0 (0%)	–	0 (0%)	0 (0%)	–	0 (0%)	0 (0%)	–	1 (17%)	0 (0%)	–	1 (17%)	0 (0%)	–	1 (17%)
Abdomen pain (severity)	0 (0%)	–	0 (0%)	0 (0%)	–	0 (0%)	0 (0%)	–	0 (0%)	0 (0%)	–	1 (17%)	0 (0%)	–	0 (0%)	0 (0%)	–	0 (0%)
Abdomen pain (interfere)	0 (0%)	–	0 (0%)	0 (0%)	–	0 (0%)	0 (0%)	–	0 (0%)	0 (0%)	–	1 (17%)	0 (0%)	–	0 (0%)	0 (0%)	–	0 (0%)
Hand-foot syndrome (severity)	0 (0%)	–	0 (0%)	0 (0%)	–	0 (0%)	0 (0%)	–	0 (0%)	0 (0%)	–	0 (0%)	0 (0%)	–	1 (17%)	0 (0%)	–	0 (0%)
Numbness/tingling (severity)	0 (0%)	–	0 (0%)	0 (0%)	–	1 (20%)	0 (0%)	–	0 (0%)	0 (0%)	–	0 (0%)	0 (0%)	–	3 (50%)	0 (0%)	–	3 (50%)
Numbness/tingling (interfere)	0 (0%)	–	2 (33%)	0 (0%)	–	1 (20%)	0 (0%)	–	0 (0%)	0 (0%)	–	3 (50%)	0 (0%)	–	3 (50%)	0 (0%)	–	2 (33%)
Dizziness (severity)	0 (0%)	–	0 (0%)	0 (0%)	–	0 (0%)	0 (0%)	–	0 (0%)	0 (0%)	–	0 (0%)	0 (0%)	–	0 (0%)	0 (0%)	–	0 (0%)
Dizziness (interfere)	0 (0%)	–	0 (0%)	0 (0%)	–	0 (0%)	0 (0%)	–	0 (0%)	0 (0%)	–	0 (0%)	0 (0%)	–	0 (0%)	0 (0%)	–	0 (0%)
Headache (frequency)	0 (0%)	–	1 (17%)	0 (0%)	–	0 (0%)	0 (0%)	–	0 (0%)	0 (0%)	–	0 (0%)	0 (0%)	–	1 (17%)	0 (0%)	–	1 (17%)
Headache (severity)	0 (0%)	–	0 (0%)	0 (0%)	–	0 (0%)	0 (0%)	–	0 (0%)	0 (0%)	–	0 (0%)	0 (0%)	–	0 (0%)	0 (0%)	–	0 (0%)
Headache (interfere)	0 (0%)	–	1 (17%)	0 (0%)	–	0 (0%)	0 (0%)	–	0 (0%)	0 (0%)	–	0 (0%)	0 (0%)	–	0 (0%)	0 (0%)	–	0 (0%)
Fatigue (severity)	0 (0%)	–	0 (0%)	0 (0%)	–	0 (0%)	0 (0%)	–	0 (0%)	0 (0%)	–	0 (0%)	0 (0%)	–	1 (17%)	0 (0%)	–	2 (33%)
Fatigue (interfere)	1 (17%)	–	1 (17%)	0 (0%)	–	2 (40%)	0 (0%)	–	0 (0%)	0 (0%)	–	2 (33%)	1 (17%)	–	2 (33%)	0 (0%)	–	2 (33%)

**Table 3:** Adverse events and intensity over 3 cycles of CAPOX in both treatment groups*

Intensity and adverse effect	Intervention *N* (%)	Control *N* (%)
Mild/moderate		
Abdominal pain	0 (0%)	1 (16.7%)
Anxiety	1 (16.7%)	0 (0%)
Belching	0 (0%)	1 (16.7%)
Chest pain	0 (0%)	1 (16.7%)
Constipation	0 (0%)	3 (50%)
Diarrhoea	2 (33.3%)	3 (50%)
Dizziness	1 (16.7%)	0 (0%)
Dyspepsia	1 (16.7%)	0 (0%)
Dyspnoea	0 (0%)	1 (16.7%)
Fatigue	6 (100%)	5 (83.3%)
Fever	1 (16.7%)	1 (16.7%)
Gall bladder pain	0 (0%)	1 (16.7%)
Gastro-oesophageal reflux	0 (0%)	1 (16.7%)
Headache	1 (16.7%)	0 (0%)
Hypotension	1 (16.7%)	0 (0%)
Infusion-related reaction	0 (0%)	2 (33.3%)
Jaw pain	1 (16.7%)	0 (0%)
Laryngospasm	0 (0%)	1 (16.7%)
Nausea	2 (33.3%)	4 (66.7%)
Paraesthesia	2 (33.3%)	2 (33.3%)
Peripheral neuropathy	5 (83.3%)	6 (100%)
Polypectomy	1 (16.7%)	0 (0%)
Right upper quadrant pain	0 (0%)	1 (16.7%)
Unsteadiness	0 (0%)	1 (16.7%)
Urinary tract infection	0 (0%)	1 (16.7%)
Severe		
Abdominal pain	0 (0%)	1 (16.7%)
Diarrhoea	0 (0%)	1 (16.7%)
Hypokalaemia	0 (0%)	1** (16.7%)
Infusion-related reaction	0 (0%)	1 (16.7%)
Jaw pain	0 (0%)	1 (16.7%)
Paraesthesia	0 (0%)	2 (33.3%)
Peripheral neuropathy	1 (16.7%)	0 (0%)
Thromboembolic event	1 (16.7%)	0 (0%)
Vomiting	0 (0%)	1 (16.7%)

^*^7 of these events caused hospitalisation (1 event in the fasting group [1 participant] and 6 in the control [3 participants])

^**^Participant with severe hypokalaemia also had life-threatening hypokalaemia

#### Adherence to intervention

In cycle 1, 100% of participants adhered to the fast. One participant stopped treatment after cycle 1; of the remaining participants, 100% adhered to the fast in cycles 2 and 3 (see Table 4).

**Table 4:** Mean values in clinical outcomes assessed at follow-up assessments for intervention and control groups

	Intervention			Control		
	Cycle 1 Day 1	Cycle 2 Day 1	Cycle 3 Day 1	Cycle 1 Day 1	Cycle 2 Day 1	Cycle 3 Day 1
*N*	6	5	5	6	6	6
Weight (kg) mean (SD)	90.7 (15.2)	91.9 (15.2)	91.8 (15.0)	79.4 (18.4)	79.4 (19.1)	79.5 (19.4)
Energy intake (kcal) (mean)	12 (27)	31 (58)	30 (59)	1816 (478)	1768 (590)	1683 (585)
Adherence	6/6	5/5^*^	5/5^*^	N/A	N/A	N/A
Average hand grip strength (kg), mean (SD)	32.6 (11.1)	Not assessed	33.0 (11.3)	23.3 (5.3)	Not assessed	25.8 (10.1)
Changes in capecitabine and oxaliplatin dose *n* (%)		3/6 (50%)	1/5 (20%)		2/6 (33.3%)	1/6 (16.7%)
Dose increased		1/6 (16.7%)	1/5 (20%)			
Dose reduced		1/6 (16.7%)			2/6 (33.3%)	1/6 (16.7%)
Treatment stopped		1/6 (16.7%)				

^*^One participant stopped treatment after cycle 1

#### Acceptability and tolerability

Ten out of the 12 participants (five in the control; five in the intervention arm) consented to an interview regarding their experiences regarding participation in SWiFT including the data collection processes. One participant declined and the second was discharged before consent could be taken.

#### Benefits/motivation

Motivation for trial participation included perceived benefits (i.e. reduction in side effects) and altruism (i.e. benefiting others in the future). Participants focused on the benefits of fasting in relation to a reduction in side effects, despite the trial’s purpose to explore whether people could fast before their chemotherapy.

*The perceived benefits were that the side-effects would be reduced. Well, as far as I’m concerned, I had little or no side-effects. Very few, you know, like frozen hands, darkened palms, I think like that. But I didn’t have any of the vomiting or extreme ones.* (Participant 08, fast)

One individual in the control group reported disappointment that they were not in the fasting arm because of the perceived reduction of side effects.

*…it literally was everything on the side effects thing apart from, as I say, I didn’t get this red rash on your skin. Apparently there are bits of your skin that can become really sensitive. I didn’t get that but I got everything else, which is why I was a bit disappointed to be in the control group. Because I think for the SWiFT trial they were testing if you fast 36 hours beforehand, would the side effects be any less.* (Participant 09, control)

#### Acceptability of fasting

The five participants randomised to the intervention arm reported adhering to the 36-h fast before each of their treatment cycles.

*I just drank plenty of water, because that’s what they suggested. Drink plenty of water, plenty of fluids. I filled the forms in when they gave them to me. You know, if you wanted a snack, you wrote it down. But I never seemed- I just had my coffees. I used to have, like, four coffees. I drink black coffee and I had four coffees a day and the rest was water, when I was fasting.* (Participant 12, fast)

*So, Monday morning I went and got my bloods done, I had a meal Monday afternoon, and then from 6 o’clock onwards, I didn’t eat until I got back from my chemo on Wednesday afternoon.* (Participant 7, fast)

Despite the 36-h fast being achievable, three of the five reported difficulties/challenges which included feeling weak/tiredness, dizziness, HCP unable to find a vein, and the fast being extended due to delays in treatment.

*And I think it was the second one, where, after fasting for 36 hours, there were a lot of delays before I actually sat and infused my chemo. So the fast wasn’t for 36 hours, it was more like 40 hours. (Laughter). So the staff could have prepared, knowing that somebody has been fasting for 36 hours, and tried to move things on, so that the person can get done and eat. I don’t know, it was the third course of my chemo and I was very irritable, extremely irritable. I’ve never known myself to react to people that way. But there was a poor nurse, I wasn’t very kind to her, put it that way.* (Participant 08, fast)

Participants described numerous side effects whilst going through CAPOX chemotherapy (e.g. pins and needle sensations, headaches, tiredness, diarrhoea, nausea/vomiting, dry mouth, loss of taste, difficulties chewing, sensitivity to the temperature of food and drink) and a couple of the participants felt that fasting was a ‘big ask’ on top of their chemotherapy regime.

*I think I just felt that, “Hey-ho, I’m going through all of this and you don’t want me to eat for 24 hours. That’s quite a big ask.” That was the way I perceived it, I think* (Participant 01, fast)

*I think there were two things. I think the first thing is that the chemotherapy was a far bigger thing than I thought it would be, so that was something that was quite a big thing to take on board. That’s number one. Then number two: I think I’d underestimated the impact of not eating anything for 24 hours. I think that that, against the background of the impact of the chemotherapy, is quite a big thing to take on board, really…* (Participant 01, fast)

*because psychologically as well, you’re not in a good place. I think in the forms, most things I was reporting regularly, for me, was anxiety. I was anxious of my finances. I was anxious of whether I was going to live. “Have I put things in order? Do my children know what to do?” Those were more of my concerns. And then in such circumstances, when you’re feeling sorry for yourself, it’s easy to feel, “Well, damn it all, who gives a…?” You know what I mean? “Am I sure I’m going to live? I might as well catch the little pleasures before I go.” (Laughter)* (Participant 08, fast)

This may be compounded by the fact that participants in the control indicated having greater appetite towards the end of their treatment cycles which coincides with the timing of the fast.

*I think, just before I was due my next cycle, I used to get a bit of appetite because I knew then I’d have to more or less fill in, whether it was psychological. I knew I had to eat more because I knew I wouldn’t be eating a lot after.* (Participant 4, control)

*The food diary I was keeping was before I took it. Because I would have been off the tablets for half a week when I started the food diary, my system and appetite and everything was basically back to normal. During the treatment my appetite and things had been a bit all over the place.* (Participant 11, control)

*After the treatment, after the infusion and maybe a week later in the cycle I was much better, I could eat more.* (Participant 3, control)

Participants reported various strategies to help adhere to the fast: walking and reading for distraction, reducing food temptations and activity levels. Previous experience of fasting was drawn upon when considering how achievable the fast would be:

*I am a Christian anyway, so fasting is part of the Christian doctrine. So I’m not new to fasting. I’ve never done 36 hours before, but this was for at least something you knew that if it could improve things, it would be better. So yes, I didn’t have much problem fasting. All I did was make sure there wasn’t much activity, just drink water, while away the day, watch TV, and things like that.* (Participant 8, fast)

*I’d been doing some kind of fasting before when I was having a series of colonoscopies, before my surgery, before the chemo. So fasting for basically two days wouldn’t be too much of an issue.* (Participant 11, fast)

Two of the participants in the fasting group described a link between believing your body requires regular food and the ability to fast.

*I could drink water, so I mean, you can last four weeks without food, but you can only last a week without water, can’t you?* (Participant 7, fast)

One of these participants felt this notion could hinder others fasting.

*Participant: But I can appreciate that there are people out there who would have found it a bit difficult, fasting.*

*Interviewer: In what way difficult do you think?*

*Participant: I don’t know, there’s lots of people who… They think they need food, don’t they? Every day, every, you know, whenever.* (Participant 2, fast)

Two of the five in the fasting group reported being determined in nature; this characteristic may have supported the fast:

*I found it very easy. I’m one of those people who you put your mind to something, you do it.* (Participant 2, fast)

Most reported a reduced appetite due to the chemotherapy side effects (e.g. nausea/vomiting, diarrhoea, dry mouth, loss of taste, difficulties chewing, sensitivity to the temperature of food and drink) which affected eating habits:

*I think your appetite is affected. I think you go off food a little bit. I think food becomes less appetising. Towards the end of the cycles, I lost my sense of taste, which I found very, very difficult.* (Participant 01, fast)

*I didn’t have an appetite. My appetite was severely reduced. I would eat a little bit of porridge in the mornings, not my normal amount, and then a little bit of maybe soup. But I really didn’t haven’t any appetite during the chemo, but I tried. I knew it was important to eat sensibly, but it was difficult to eat at times. Just looking at food is like, “Oh, I don’t want it”.* (Participant 03, control)

*…because of the side effects that I had, it was very limited. I had a tingling in my throat. It was like razor blades, if I ate anything cold.* (Participant 04, control)

*I think I eat less when I’m on it. I’m less likely to finish a plate of food because it’s there. More likely to just stop when I’m done because the food doesn’t taste as good, it’s not as enjoyable. So I’m less likely to eat more, I guess.* (Participant 11, control)

One person spoke of this reduction in appetite having supported their ability to fast and how they felt the fast could have been extended to 48 h.

*I found it really quite straightforward. You see, the thing is, with me, I’ve had no appetite* (Participant 12, fast)

Others who fasted felt that any longer would have been difficult and one participant felt that a 24-h fast would be more appealing.

#### Study materials

Overall, participants experienced the trial material to be ‘straightforward’ to complete with the exception of one participant who felt the questionnaires were tedious and repetitive and two participants who felt the appetite question was difficult to answer:

*Although the one that I thought was a bit strange was your appetite. I never knew what to put. I think I ended up putting 50% because I’m thinking, “Well, you wouldn’t know. If you were absolutely normal and taking nothing, what would you put then? Because you are not hungry all the time.” I found that question just a little bit confusing* (Participant 09, control)

Support and advice were dependant on the participant contacting the trial team and one participant would have liked the team to initiate contact.

*I think what would have been helpful during the course of this chemotherapy regime over three months is for you to have two or three phone calls, perhaps partway through each cycle: “How are you doing? How are you getting on?” instead of that you had to initiate that exchange. Contact was great and the advice was great, but it was incumbent on the patient, on the recipient of the chemotherapy, to get in touch if there were any issues going on.* (Participant 01, fast)

#### Factors affecting recruitment at study sites

Five semi-structured interviews were conducted with site team members of SWiFT: two at site A (consultant and trial co-ordinator [TC]) and three at site B (consultant, TC, and research nurse). The factors affecting recruitment are presented within four main headings: research environment related, design related, recruiter related, and participant related together with illustrative quotations, a summary of which can be found in Table 5.

**Table 5:** Factors affecting recruitment to SWiFT across 2 sites

Themes	Sub-themes	Supportive quote	Site A	Site B
Research environment related	- Concurrent competing trials	We have another trial that we get people at the same time as their study, and they will only offer us the patients that aren’t eligible for theirs, because they don’t want to overwhelm them. So, it could be a combination that their study was involving a lot of quality of life questionnaires etc., and so they just wanted us to steer clear of all of those, but also it could just be down to patient choice of they maybe just think one study is enough for them (Site A—TC) Interviewer: And I’ve also heard that there were other trials that were recruiting at the same time Respondent: Yes Interviewer: Would those have potentially slowed recruitment to SWiFT? Respondent: Don’t think so. (Site A—consultant) the biggest thing about this trial is obviously it’s offered to patients just as they are getting their first line treatment. Obviously often those patients haven’t really got any other options for first line, because we have already got the standard of care there. (Site B—TC) …there isn’t much going on. It’s always the same old treatment…It’s only Oxaliplatin and Capecitabine that anybody really uses nowadays…And there isn’t any other trials, so it is a bit of a niche here. (Site B—consultant)	P	
	- COVID • Paused for recruitment • Delay in reopening site • Lag in opening and recruitment • Recruiters seconded • Remote clinics/working instead of onsite • Staff loss/high turnover • SWiFT less priority than active treatment trials	because it was, obviously, closed for a long time. I think if we’d have, maybe, had the full time and not had any of the problems with COVID, we would have had a wider population of people (Site A—TC) When COVID first hit recruitment was stopped for every trial because we didn’t know how COVID was going to affect cancer patients. That was the main reason, and so then a lot of research staff were redeployed to help other areas. Obviously staff were off with COVID as well, so we were utilised in other areas. (Site B—RN) We had a couple of staff leave, as well. Staff turnover was quite high during the pandemic, which I think is probably the same everywhere in the hospital, but it just meant that we didn’t get to reopen the trial. (Site A, TC) Even though we were reopening things, people were still being seconded, but also, although we managed to reopen things, it was a little bit hilly because we would reopen and think we were coming down from it, and then there’d be another spike. The hospital would change its regulations, or there’d be another wave of vaccine trial recruitment that we needed to send nurses to help with, that kind of thing. It was quite up and down for a long time (Site A, TC) …and effectively during the lockdown, the other thing was you have to remember all clinical trials stopped. So, we stopped running clinical trials during the lockdown. (Site A, consultant) We probably had a period of about two months, March, April, May into 2020, when we really didn’t recruit very well into anything. But then it quickly came back with SWiFT after that, I think. (Site B, consultant) A lot of our nurses, obviously, were seconded, and they had to help out on the wards, etc. Also just the vaccine trials, as well, took up a lot of our time. We had a couple of staff leave, as well. Staff turnover was quite high during the pandemic, which I think is probably the same everywhere in the hospital, but it just meant that we didn’t get to reopen the trial (Site A, TC) Consultant: But at first, it got- there was quite a lag in the things were open and actually recruiting again Interviewer: Okay. Okay, so, just because something had reopened, it didn’t mean that there was full screening, recruitment going on at that point Consultant: That’s right (Site A, consultant) I think, in general, our trials did suffer from the consultations being remote, because patients would have had to have come in for more extra visits. If they were already in clinic anyway, it’s easier to catch them, explain the trial to them and then bring them in, whereas, if they’re remote, you then have to have an extra consultation, almost, to explain it to them, because it’s a lot harder to explain a trial remotely than it is to show them (Site A, TC) The top was a drug trial that meant that the patients who didn’t have another option would have an option if their only treatment option was that. I think, in terms of observational trials, a lot of them tended to include coming in extra. I don’t think SWiFT necessarily included that, but I think a lot of observational trials were banded together as a lower priority because they weren’t necessarily directly beneficial to the patients. At the time, then they wanted to reopen the portfolio so they could benefit as many patients as possible. (Site A, TC)	P	P
Design related	+ Recruitment compatible with routine clinical practice: straightforward design and low impact data collection	It was a straightforward trial. So, it wasn’t like some of our interventional trials, which need a lot of radiology, and things like that. (Site A, consultant) so I think it’s straightforward and it’s easy to explain to people (Site A, TC) Some of them take minutes to go through, like the SWiFT one is actually pretty quick. But then others will be, the nurses or researchers or somebody will be fiddling around for an hour doing the electronic PROM’s forms and the blood tests and all things like that… the SWiFT trial is one of the really easy ones, so I think you could do it in any centre (Site B, consultant) It’s quite a nice, simple study, isn’t it, as well. I’ve worked in research before, so, yes, it was a nice one for me to pick up really (Site B, RN) I think the information sheet was very good (Site B, consultant)	P	P
	+ Realistic recruitment target (big pool of patients to recruit from—enough eligible patients; eligibility criteria not too narrow)	Consultant: We predominantly serve the [Place] area; a population of about a million, that we get regional referrals from throughout the [Place], for specialist techniques Interviewer: Hmm. So, you’re a large centre? Consultant: Yes. We’re one of the largest Interviewer: Would you say that 15 would have been a realistic recruitment estimate at your site? Respondent: Yes. (Site A, consultant) The inclusion criteria were straightforward in the sense that a lot of our trials you have to tick lots of boxes, or there are so many different other factors that mean they would be ineligible, whereas it was very straightforward to get people on (Site A, TC) We have the advantage of lots of patients and a lot of staff to see them (Site B, consultant) I think the inclusion/exclusion doesn’t seem to have pushed really anyone off the trial other than those few people we had at the beginning (Site B, TC) There are plenty of new people that come into these clinics every week or every other week…we are a big site with a big cohort of patients (Site B, TC)	P	P
Recruiter related	+ Engagement	Interviewer: what was the engagement like from your colleagues in screening for SWiFT? Were the consultants interested in the trial? Respondent: I think ‘inconsistent’ is the answer to that Interviewer: Okay. And why, what was the inconsistency down to? Respondent: Some colleagues will have been interested, and others won’t Interviewer: Is that quite normal, or is that because of SWiFT? Respondent: I think in general when they’re therapeutic trials, there’s probably broader agreement than there is for a trial such as SWiFT (Site A, consultant) …we did, I think it was mainly me, but the others were keen and were looking for patients and the nurses were keen. Nobody had any issue with it really. (Site B, consultant) Interviewer: I wondered maybe if we started with describing what your role was in SWiFT? Respondent: Well, I, sort of, sat back. I think the research nurses did what they could, basically (Site A, consultant)		P
	- High staff turnover	I think, widespread, a lot of people decided during COVID that they didn’t actually like what they was doing. (Laughter) So, we’re still suffering the staff losses due to that, where people are still deciding, “Actually, maybe this isn’t what I want to do for the rest of my life,” kind of thing. So, yes, we’re struggling in that sense as well. (Site A, TC) I think just before I started we, the team, weren’t recruiting as much because there were a lot of changes in the team in terms of the nursing side, so I think maybe certain trials got prioritised due to staffing. There might have been a little lull before I started, and then we had two or three new starters at one time not long after me, so we could then push recruitment a bit more again (Site B, RN) I think obviously we had a few staff changes at different points between the nurses, so picking up the trial perhaps meant there was a bit of a gap where potentially patients could have been missed. I think that was something that potentially halted recruitment a bit. (Site B, TC)	P	P
	- Fasting is an unknown intervention	I think that this is a really interesting trial. I’ve not really considered it or heard of it in the wider research, cancer research area so far. (Site B, TC) I think, being completely honest, a lot of people, as soon as they hear ‘fasting’, are put off. So, I think, because I don’t really know much about it, I don’t necessarily know the need, but I think, maybe, people need more education on why there’s a need, rather than just thinking, “Gosh, I don’t know if I’d want to fast.” I think sometimes that, even if the trial is valuable, if you don’t necessarily believe in it much, you might not be as good at selling it, even if it’s subconsciously. I don’t know if, maybe, that was part of the reason that we didn’t recruit. (Site A, TC)	P	P
	+ Reminders	We’re always pushing any trials that we’ve got open, just to keep people aware. Obviously, if they’ve been open a while they can go to the back of people’s minds. I think the team is actually quite good at that, if we feel like we’ve fallen behind in recruitment in certain studies we’ll bring it up at the team meeting, which is once a month, which our consultants will be on. (Site B, RN) …we did send around a few emails from time to time just to all the colorectal consultants just to remind them the study is obviously ongoing. It’s obviously a feasibility trial so it is perhaps maybe a bit, I would say, not forgotten about but obviously you have got other big trials that are going on. We did send those reminders just to keep in mind any new patients coming on board that this trial is an option for them to join, and to mention it to them. I think after that the consultants were like, “Oh okay. Yes. Don’t worry. We are remembering SWiFT is a trial. We are thinking about it for any new patients.” I think those reminders throughout the recruitment process were good. (Site B, TC)		P
	+ Active leadership	So the fact that I probably was very positive for it did help the trial. (Site B, consultant) I think that this is a really interesting trial. I’ve not really considered it or heard of it in the wider research, cancer research area so far. I know that our PI was very keen on it. (Site B, TC)		P
	+ Communication: engagement with trial and framing of trial to participants	…that’s something I did find really interesting when I read the protocol, you know, the theory behind it and why it’s been done. It was actually very interesting to me, something that I’d not been informed of before about fasting and how that can affect cells and how they work, so that was really interesting to me actually. That’s always helpful when you pick up new trials, that if you’re interested and think there’s a good reason behind it. It encourages you to approach patients and be enthusiastic about it with patients, which obviously then encourages them to join as well if you’re enthusiastic about it, I think. (Site B, RN) I think it’s the way you put it. I think if you try and put it positively and try and – if you just said, “Look, you’ve got to fast for 36 h and you can’t do this, can’t do that,” probably people would say no. But if you do it in the sort of way I said it, that you come along, you can eat Monday, you’ve got to fast Monday night, Tuesday and you can stop if you want to, then most people seemed to enter it. (Site B, consultant)		P
Participant related	- Fasting: additional burden on top of treatment	The reason people said to us why they didn’t want to go in it, was usually, I suppose there’s that bit that can’t be bothered, there’s a lot going on generally. Or the fact that they don’t want to fast. (Site B, consultant) I’d say the main issue was patients are obviously very anxious about their treatment anyway and felt like it was an added stress and added anxiety whether they’d have to fast or not. (Site B, RN) Interviewer: …when you were speaking to patients, why do you think the patients declined the study? Consultant: I think maybe of them felt they had other things to worry about, probably. I think that’s the difficulty, really. (Site A, consultant) I suppose those people personally know how they cope with being hungry. I know personally I don’t cope very well. If you’ve got that extra feeling hungry or irritable, some people get anxious when they’re hungry, that on top of having the stress of going through treatment. If they have got family, young families, would probably be too intense. It’s the impression that I got from patients who declined. (Site B, RN) I think, maybe, some patients are probably thinking, “There’s already enough going on. I’m already in a bad place. Why would I want to not eat?” because a lot of people find eating quite comforting. So, I don’t know if, maybe, that was part of it. (Site A, TC) Well I think certainly it’s feasible, definitely. Because I was worried at the beginning that nobody would want to fast (Site B, consultant)	P	P
	Length of fast considered achievable	It’s not a long time 36 h, because really you don’t eat Monday night. So we saw them Monday, treated Wednesday, so you don’t eat Monday night. Then it’s only really one full day. Then you get up and come in the next day and you have your chemotherapy and then you can eat. So it’s not a huge amount to ask anybody really on this really, I don’t think. (Site B, consultant)		

Key:—= challenge to recruitment; +  = supported recruitment

Both sites considered SWiFT to be a ‘straightforward’ trial with low impact data collection. All healthcare professionals considered the target of 15 to be realistic as they had large patient pools to recruit from and the eligibility criteria were not too narrow. Despite this, site A did not recruit to the trial and site B recruited 12 participants. The main reasons identified by the site teams for recruitment failure were the delays and disruptions caused by COVID-19 restrictions including pausing recruitment, delay in reopening, a lag in opening and recruitment commencing, a change from onsite to remote clinics, high staff turnover and loss (e.g. recruiters seconded to COVID trials), and active treatment trials taking priority. Healthcare professionals reported that a further challenge to recruitment was participants’ feeling that the fast was an additional burden on top of their standard treatment. Another challenge which was identified by the study team when setting up this trial was a possible lack of clinical equipoise (i.e. uncertainty within the clinical community) regarding fasting. Nine potential study sites were identified and approached and asked to consider being a recruiting site for SWiFT. Seven of these responded; two agreed to open the trial and five declined. Four of the five declining sites offered reasons for non-participation which were all surrounding the fast: concerns that the fast was too long (*n* = 2), not wanting to ask patients to fast (*n* = 1), and that it would be a difficult trial to recruit to (*n* = 1). Healthcare professionals were unaware of evidence relating to this area of research and there were no accounts of engagement with this topic and an uncertain level of interest within the wider community. At site B, several factors were reported to support recruitment: the lack of concurrent competing trials, recruiter-related engagement, sending reminders to recruiters, active leadership, and positive communication (see Table 5).

### Secondary outcome measures

The secondary outcome measures aim to provide further information on possible outcomes of interest in a definitive trial. These are as follows.

#### Side effects of chemotherapy

Measured using the Patient-Reported Outcomes version of the Common Terminology Criteria for Adverse Events (PRO-CTCAE™) [25], full blood count (FBC), and blood chemistry analysis. Patient-reported side effects were collected on day 1 of each cycle prior to CAPOX administration then as a follow-up on day 7, to capture the transient nature of side effects. Data were recorded on whether participants completed their first 3 cycles of chemotherapy and reasons for dose reductions/delays/early termination if they occurred (Tables 2 and 3).

#### Quality of life

Measured using the EQ-5D-5L health-related quality of life instrument [26] (Table 2).

#### Haematologic toxicities

Assessed using routine FBC data collected prior to each round of chemotherapy and classified according to CTCAE criteria [27] (Table 2).

#### Markers of cellular metabolism

**Fig. 2:**
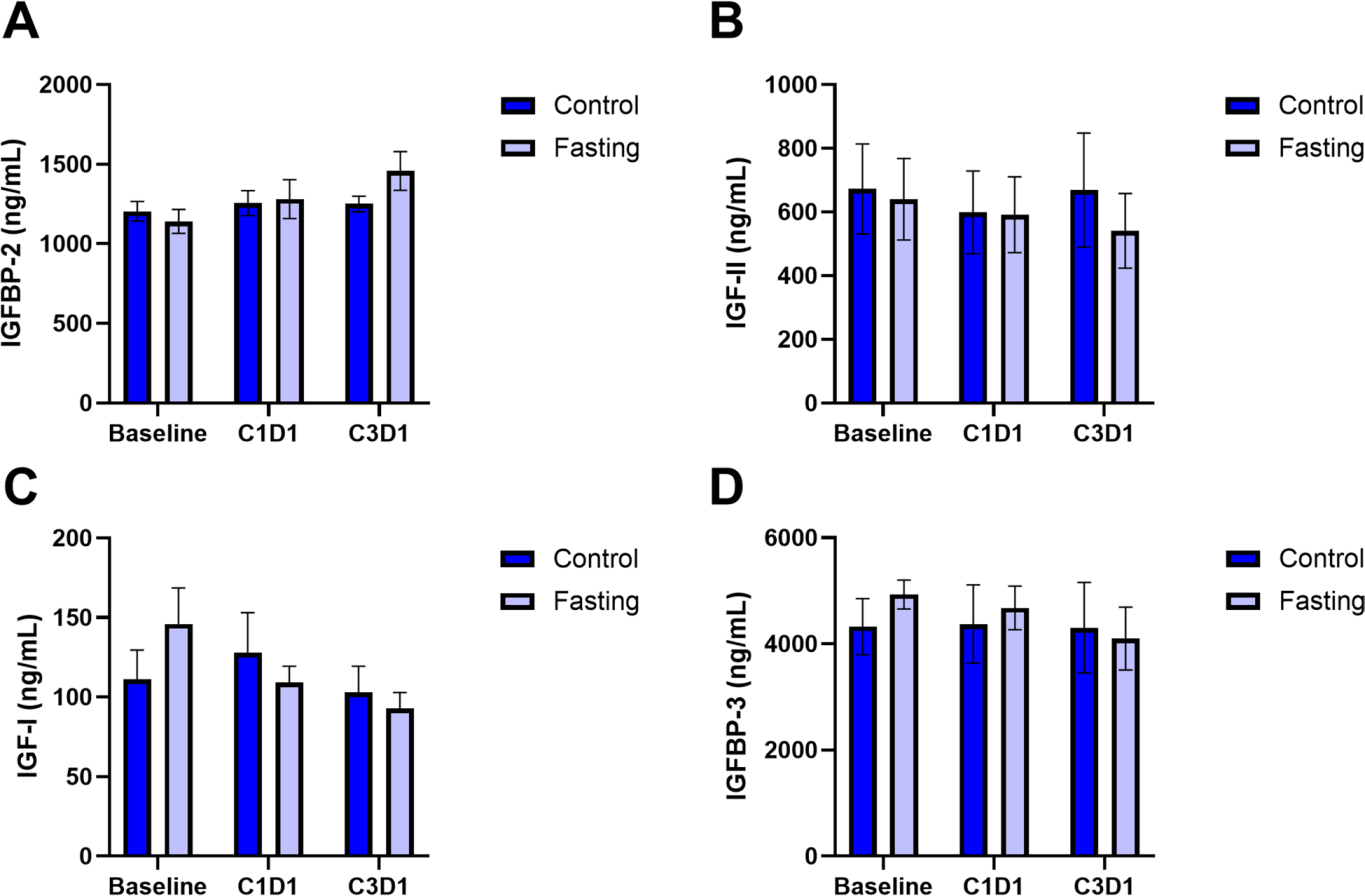
Mean serum concentrations of IGFBP-2, IGF-II, IGF-I, and IGFBP-3. Levels of IGFBP-2 (**A**), IGF-II (**B**), IGF-I (**C**), and IGFBP-3 (**D**) were measured in control and fasting groups prior to fasting (baseline) and post 36-h fasting (Cycle 1, Day 1 (C1D1) and Cycle 3, Day 1 (C3D1)). *N* = 5 participants per group. Error bars represent SEM

Following both 36-h fasts where IGFs were measured (i.e. cycles 1 and 3), IGFBP-2 levels increased in the fasting group compared to baseline (Fig. 2A). IGF-II levels did not change in either the control or fasting groups at the different time points (Fig. 2B) but, as anticipated, there was a noticeable trend towards a reduction in both IGF-I and IGFBP-3 (Fig. 2C and D, respectively) following fasting compared to baseline.

#### Markers of inflammation

C-reactive protein (CRP) was measured at baseline (pre-fast) and prior to chemotherapy administration at cycles 1 and 3. See Table 6 for results. Four of the six values were < 5 mg/L for CRP and therefore the mean could not be calculated. Individual values are, however, listed in the footnote.

**Table 6:** Markers of inflammation and cellular metabolism

Mean (SD)	Intervention				Control			
	Baseline	Cycle 1 Day 1	Cycle 2 Day 1	Cycle 3 Day 1	Baseline	Cycle 1 Day 1	Cycle 2 Day 1	Cycle 3 Day 1
CRP (mg/L)	N/A*	N/A*		N/A*	N/A*	N/A*		N/A*
Glucose (mmol/L)	5.9 (1.2)	5.4 (1.2)		6.4 (1.1)	5.4 (0.5)	5.6 (0.5)		5.5 (0.9)
Insulin (mIU/mL)	85.8 (57.4)	37.8 (29.0)		46.0 (27.4)	75.4 (95.6)	N/A*		44.0 (46.5)
Haematology								
Red blood count (1012/L)	5 (0.3)		4.8 (0.3)	4.7 (0.5)	4.3 (0.5)		4.2 (0.3)	4.2 (0.3)
Haemoglobin (g/L)	141.7 (6.5)		137.5 (6.6)	137.8 (8.4)	132.0 (12.3)		131.5 (8.9)	13.27 (4.8)
Neutrophils (109/L)	4.6 (1.5)		3.7 (1.1)	2.7 (1.3)	3.5 (1.2)		2.7 (0.7)	2.6 (0.6)
Lymphocytes (109/L)	2 (0.7)		1.9 (0.6)	1.5 (0.5)	1.8 (0.6)		1.8 (0.7)	2.1 (0.5)
Monocytes (109/L)	0.4 (0.2)		0.5 (0.2)	0.4 (0.1)	0.3 (0.1)		0.5 (0.1)	0.4 (0.2)
Eosinophils (109/L)	0.2 (0.1)		0.3 (0.2)	0.2 (0.3)	0.2 (0.3)		0.2 (0.2)	0.1 (0.1)
Basophils (109/L)	0.1 (0.1)		0.0 (0.0)	0.0 (0.1)	0.1 (0.1)		0.1 (0.1)	0.0 (0.1)
Platelets (109/L)	293.2 (78.8)		230 (62.9)	208.0 (87.9)	271.5 (37.5)		272.5 (61.9)	242.5 (34.4)
Liver function								
ALT (IU/L)	24.3 (7.5)		23.7 (4.5)	45.3 (5.1)	26.3 (10.9)		27.0 (12.6)	32.0 (8.5)
AST (IU/L)	23.5 (1.9)		25.5 (2.6)	42.8 (6.9)	29.3 (12.2)		31.8 (10.1)	32.2 (2.9)
Bilirubin (µmol/L)	7.5 (2.3)		8.0 (1.1)	9.2 (3.3)	9.8 (5.8)		10.2 (5.0)	13.5 (10.3)
ALP (IU/L)	75.8 (17.8)		75.2 (18.1)	76.0 (22.6)	109.2 (84.0)		101.0 (60.3)	101.2 (51.2)
Albumin (g/L)	46.3 (2.0)		43.7 (1.8)	42.0 (2.8)	46.0 (3.2)		43.3 (1.6)	42.3 (1.5)
Total protein (g/L)	72.0 (3.7)		67.3 (2.4)	66.6 (5.8)	71.8 (3.8)		57.0 (25.2)	66.0 (2.6)
Urea and electrolytes								
Sodium (mmol/L)	141.2 (2.5)		141.0 (1.1)	141.6 (2.3)	141.3 (1.5)		141.7 (1.6)	141.2 (1.7)
Potassium (mmol/L)	4.5 (0.2)		4.4 (0.5)	4.3 (0.1)	4.6 (0.3)		4.6 (0.4)	4.4 (0.2)
Creatinine (μmol/L)	89.3 (28.7)		81.7 (18.7)	84.8 (20.8)	62.3 (17.9)		66.8 (13.4)	67.3 (15.2)
Urea (mmol/L)	5.8 (2.9)		4.4 (1.4)	4.1 (1.6)	4.9 (1.0)		4.5 (1.2)	4.8 (0.9)
IGFs								
IGF-I (ng/mL)	145.9 (50.6)	109.1 (22.8)		92.8 (22.5)	111.3 (40.8)	127.8 (56.5)		102.9 (37.0)
IGF-II (ng/mL)	640.0 (286.5)	591.0 (266.7)		540.8 (261.9)	672.5 (316.5)	598.6 (290.0)		668.7 (400.1)
IGFBP-2 (ng/mL)	1140.8 (166.1)	1280.8 (270.9		1457.8 (271.1)	1204.6 (137.5)	1256.1 (173.9)		1250.2 (111.1)
IGFBP-3 (ng/mL)	4931.8 (610.5)	4678.1 (913.6)		4099.7 (1322.6)	4326.6 (1178.2)	4373.7 (1647.9)		4304.3 (1903.6)

^*^Means could not be calculated for these variables. Results for individual participants were as follows:

CRP (mg/L) intervention at baseline: < 5, < 5, 0, 0, 16.2, 0

CRP (mg/L) control at baseline: < 5, 33.9, < 5, 0, 0, 0

CRP (mg/L) intervention at Cycle 1 Day 1: < 5, < 5, < 5, not determined, 8.4, < 5, 0

CRP (mg/L) control at Cycle 1 Day 1: < 5, 6.7, 0, 0, 0, not determined

Insulin (mIU/mL) control at Cycle 1 Day 1: < 10, 32, 26, 16, 360, not determined

CRP (mg/L) intervention at Cycle 3 Day 1: < 5, < 5, < 5, < 5, < 5

CRP (mg/L) control at Cycle 3 Day 1: < 5, 8, 0, < 5, < 5, < 5

#### Appetite

Self-reported on visual analogue scales (VAS) [28]. See results in Table 2.

## Discussion

This is the first study to have assessed the feasibility of short-term water-only fasting at the time of chemotherapy for colorectal cancer. A total of 12 participants were recruited to the study, and all but one remained in the trial for the first three cycles of their chemotherapy schedule. Despite toxicities accumulating over a number of cycles, participants reported adhering to the fast. In addition, participant retention and data completion rates were good. This study indicates that fasting at the time of chemotherapy is a feasible intervention.

SWiFT was purposefully designed to be a pragmatic trial that closely followed the usual clinical pathway, and aimed to avoid additional clinic visits for patients and to limit, as far as possible, the number of additional assessments and procedures that needed to be undertaken. SWiFT achieved this as healthcare professionals considered SWiFT to be a straightforward trial. Despite the small sample size, the trends observed for IGF-I and IGFBP-2 and IGFBP-3 were consistent with effective fasting and these data may help to inform sample size calculations in future trials. A larger trial would confirm the trends observed in components of the IGF axis and would also consolidate all the secondary outcome measures that were considered in this feasibility trial.

Participant recruitment was severely impacted by the COVID-19 pandemic, which resulted in the trial being suspended at both study sites and substantial delays to the start of recruitment. Furthermore, the reduced capacity at sites (due to added pressures on NHS staff time resulting from the COVID-19 pandemic) when studies were able to re-open meant that recruitment continued to be impacted throughout. The target sample size was 30, but only 12 participants were recruited and all from one study site. In order to explore potential reasons for the inability to recruit participants from one of the study sites, we conducted interviews with site staff involved in recruitment. Given the potential for fasting or other forms of dietary restriction to improve outcomes of chemotherapy treatment, future studies should invest time at the set-up stage to ensure full engagement and education regarding the theoretical rationale for fasting which may improve recruitment as this is still a novel intervention.

Strengths of this study include the fact there was an embedded qualitative study and interviews with participants and healthcare providers involved in trial delivery were conducted. However, it is unfortunate that we were only able to interview two individuals from the study site that was not able to recruit any participants, resulting in relatively scant data on reasons why this was the case.

One of the main limitations, however, is that the number of people recruited was relatively low. Sample sizes of around 30 are usually recommended for pilot or feasibility studies [17], but only 12 people were recruited to SWiFT. Nonetheless, all were recruited from one study site where the target sample size was 15 participants.

The stark contrast between the two study sites with regard to recruitment means that it is difficult to determine the true recruitment rate. The overt (e.g. suspension of recruitment to non-COVID-19 trials) and covert (e.g. reluctance to attend screening appointments, thereby potentially reducing the patient pool) effects of the COVID-19 pandemic certainly had a major impact on recruitment, but other factors such as concurrent competing trials, high staff turnover, and the intervention being considered an additional burden may also have played a role.

## Conclusion

These findings suggest that short-term water-only fasting at the time of chemotherapy can be a feasible intervention. However, recruitment may be challenging and success in recruitment may depend more on specific local factors rather than the intervention being unattractive to most potential participants.

## Supplementary Information

Supplementary Material 1.

## Data Availability

The dataset(s) supporting the conclusions of this article are included within the article.

## Abbreviations

CAPOX: Capecitabine and Oxaliplatin
SWiFT: Short-term, Water-only Fasting Prior to Chemotherapy Trial
IGF: Insulin-like growth factor
IGFBP: Insulin-like growth factor binding protein
DSR: Differential stress resistance
RCT: Randomised controlled trial
BMI: Body mass index
MDT: Multidisciplinary team
BMR: Basal metabolic rate
CT: Computerised tomography
CRP: C-reactive protein
TC: Trial co-ordinator
PRO-CTCAE™: Patient-Reported Outcomes version of the Common Terminology Criteria for Adverse Events
FBC: Full blood count
VAS: Visual analogue scales
